# HIV-1 gp120 Impairs Spatial Memory Through Cyclic AMP Response Element-Binding Protein

**DOI:** 10.3389/fnagi.2022.811481

**Published:** 2022-05-09

**Authors:** Jenny Shrestha, Maryline Santerre, Charles N. S. Allen, Sterling P. Arjona, Carmen Merali, Ruma Mukerjee, Kumaraswamy Naidu Chitrala, Jin Park, Asen Bagashev, Viet Bui, Eliseo A. Eugenin, Salim Merali, Marcus Kaul, Jeannie Chin, Bassel E. Sawaya

**Affiliations:** ^1^Molecular Studies of Neurodegenerative Diseases Lab, Philadelphia, PA, United States; ^2^Fels Cancer Institute for Personalized Medicine Institute, Philadelphia, PA, United States; ^3^Department of Pharmaceutical Sciences, School of Pharmacy, Temple University, Philadelphia, PA, United States; ^4^Memory and Brain Research Center, Department of Neuroscience, Baylor College of Medicine, Houston, TX, United States; ^5^Department of Neuroscience, Cell Biology, and Anatomy, The University of Texas Medical Branch, Galveston, TX, United States; ^6^Infectious and Inflammatory Disease Center, Sanford Burnham Prebys Medical Discovery Institute, La Jolla, CA, United States; ^7^Department of Psychiatry, University of California, San Diego, San Diego, CA, United States; ^8^Division of Biomedical Sciences, School of Medicine, University of California, Riverside, Riverside, CA, United States; ^9^Department of Neurology, Lewis Katz School of Medicine, Temple University, Philadelphia, PA, United States; ^10^Department of Cancer and Cell Biology, Lewis Katz School of Medicine, Temple University, Philadelphia, PA, United States; ^11^Department of Neural Sciences, Lewis Katz School of Medicine, Temple University, Philadelphia, PA, United States

**Keywords:** HIV, neurodegeneration, CREB protein, mitochondria, rolipram

## Abstract

HIV-associated neurocognitive disorders (HAND) remain an unsolved problem that persists despite using antiretroviral therapy. We have obtained data showing that HIV-gp120 protein contributes to neurodegeneration through metabolic reprogramming. This led to decreased ATP levels, lower mitochondrial DNA copy numbers, and loss of mitochondria *cristae*, all-important for mitochondrial biogenesis. gp120 protein also disrupted mitochondrial movement and synaptic plasticity. Searching for the mechanisms involved, we found that gp120 alters the cyclic AMP response element-binding protein (CREB) phosphorylation on serine residue 133 necessary for its function as a transcription factor. Since CREB regulates the promoters of PGC1α and BDNF genes, we found that CREB dephosphorylation causes PGC1α and BDNF loss of functions. The data was validated *in vitro* and *in vivo*. The negative effect of gp120 was alleviated in cells and animals in the presence of rolipram, an inhibitor of phosphodiesterase protein 4 (PDE4), restoring CREB phosphorylation. We concluded that HIV-gp120 protein contributes to HAND via inhibition of CREB protein function.

## Introduction

Patients infected with HIV-1, including those using combinatory antiretroviral therapy (cART), suffer from deregulation and impairment of organs such as the heart, kidney, and brain (Fellows et al., 2014). Studies unequivocally link HIV-1 infection and neurocognitive disorders such as spatial memory impairment and learning disability (SMI-LD) (Goodkin et al., 2017). These disorders may be caused by the release of viral proteins from defective proviruses, or integrated HIV-1 incapable of producing infectious virions due to mutations in the open reading frame yet still capable of producing viral proteins, which explains the detection of viral proteins such as Tat and gp120 in CSF (Bruner et al., 2016; Imamichi et al., 2016; Burbelo et al., 2018; Kuniholm et al., 2021; Siliciano and Siliciano, 2021; Cho et al., 2022). Hence, a significant number of HIV-1 patients suffer from SMI-LD.

HIV-1 gp120 is the envelope protein that permits the association of the virus with CD4+ cells including T cells, and cells of the myeloid lineage (macrophages and microglia). Though HIV-1 has not been shown to directly infect neurons, viral proteins released by infected cells influence neurons. gp120 can directly interact with receptor CXCR4 to gain access to the cell by lipid-raft mediated endocytosis or pinocytosis (Berth et al., 2015). In neurons, gp120 protein: (i) causes an increase in calcium influx, (ii) activates the oxidative stress (OS) pathway, and (iii) alters mitochondrial functions and the release of toxic lipids (Avdoshina et al., 2016; Mocchetti et al., 2012; Fields et al., 2016a,b; Rozzi et al., 2017; Fields and Ellis, 2019; Zhang et al., 2019). Knockout or knockdown of CCR5 protects against gp120 V3 peptide-induced memory deficits (Maung et al., 2014; Zhou et al., 2016). We recently demonstrated that gp120 protein promotes metabolic reprogramming that contributes to mitochondria loss of energy and movement.

*Per* the literature, studies describe the cAMP response element-binding protein (CREB) as the regulator of mitochondrial biogenesis through the regulation of the proliferator-activated receptor gamma coactivator (PGC) 1α promoter (Shi et al., 2005; Wu et al., 2006; Chowanadisai et al., 2010; Liu et al., 2014).

Cyclic AMP response element-binding protein was first described as a cAMP-responsive transcription factor regulating the somatostatin gene (Montminy and Bilezikjian, 1987). It binds to DNA sequences called *cAMP response element* (CRE) (Carlezon et al., 2005; Altarejos and Montminy, 2011), thereby increasing the transcription of the downstream genes such as BDNF (Tao et al., 1998), Bcl-2 (Meller et al., 2005), c-fos (Tu et al., 2013), Mitochondrial Pyruvate Carrier 1 (MPC1) (Lou et al., 2019), and interleukin (IL)-10 (Sanin et al., 2015).

Cyclic AMP response element-binding is phosphorylated on various residues; however, its phosphorylation on serine residue 133 by protein kinase A (PKA) and other kinases is critical for binding to CRE motifs (Johannessen and Moens, 2007). CREB plays a role in the regulation of short- and long-term memory formation (Bourtchuladze et al., 1994). It is essential to the formation of spatial memory (Mizuno and Giese, 2005) and plays a positive role in axonal transport and synaptic plasticity (Abel and Nguyen, 2008; Lin and Holt, 2008). In the adult brain, CREB is involved in learning, memory, and neuronal plasticity. BDNF is an essential gene for synaptic plasticity, memory consolidation, and long-term potentiation (Suzuki et al., 2011). Therefore, inhibition of CREB protein will have multiple damaging outcomes contributing to spatial memory impairment (Santerre et al., 2019).

The role played by phosphorylated CREB protein in spatial memory provided the rationale to determine whether gp120 impairs spatial memory through CREB?

## Materials and Methods

### Cell Culture, Treatments, and Transfection

The human neuroblastoma cell line, SH-SY5Y, was purchased from ATCC (CRL-2266) and grown in DMEM F12 supplemented with 10% FBS, 1% non-essential amino acid, and 1% sodium pyruvate. The cells were incubated at 37°C supplemented with 5% CO_2_ and passed each time at 85–90% confluency. The cells only within 10 passages from the time of purchase were used. The cells were seeded at the density of 5 × 10^5^ cells/per well in 6 well plates. They were differentiated into neurons with 10 μM retinoic acid (RA) treatment for at least 3-4 days.

HEK293T cells were purchased from ATCC (CRL-1573) and grown in DMEM supplemented with 10% FBS. All cells were incubated at 37°C supplemented with 5% CO_2_. The cells were passed each time at 85–90% confluency. The cells were seeded at the density of 5 × 10^5^ cells/per well in 6 well plates.

Human primary neuronal cells (26 weeks old fetal brain) were generously provided by Dr. Eliseo Eugenin. The human cortical fetal tissue was used to isolate the mixed culture of neurons and astrocytes as described (Orellana et al., 2014). The culture obtained contained 30–40% neurons, 60–70% astrocytes, and 2–5% microglia. Neuronal enriched cultures were obtained after 7–10 days of culture in a Neurobasal medium supplemented with N2 neuro-survival factor and 5% FBS. The cells were cultured for about 10 days to obtain 70–80% of the neuronal culture before the treatments. (*Human primary culture experiments were performed before 2016*).

Primary C57B1/6J mouse neurons were obtained from E18 (embryonic day 18). The cortex and hippocampus were separated and rinsed in HBSS before digesting in 0.125% trypsin. The cortex and hippocampus were digested for 15 and 20 min, respectively (DNase 0.4%). The digested tissue was triturated in DMEM containing 10% FBS followed by centrifugation at 1000rpm for 10 min. The tissue was then washed twice, resuspended in DMEM F12, and passed through a strainer to remove the non-dispersed tissue. The cells were seeded at 7 × 10^5^ cells/well in 6-well plates and 1.2 × 10^5^ cells/chamber in 4-chamber slides and incubated at 37°C supplemented with 5% CO_2_. DMEM medium was replaced with Neurobasal supplemented with B27, NEAA, GluMax, and pen-strep after 2–3 h of seeding. Half of the medium was changed every 3 days.

For transient transfection assay, 1 × 10^5^ of cells were transfected using lipofectamine 2000 with 0.5 μg of pcDNA_3_ (Invitrogen™ #V79020), or pcDNA_3_-CREB expression plasmid (Addgene #22394). The cells were differentiated 24 h post-transfection and then treated with gp120 protein.

### HIV-1 gp120 Treatments

Recombinant HIV-1_IIIB_ gp120 (clade B), HIV-1_96ZM651_ gp120 (clade C), and HIV-JR-CSF-Fc-gp120 proteins were kindly received from NIH AIDS Reagent Program. Fc-gp120 chimera was constructed by fusing human IgG1 Fc domain N-terminal to gp120 from HIV-1 JR-CSF. Fc was fused in-frame to the Leu residue at position 51 (Binley et al., 2006). Samples were treated for 24 or 48 h in concentration indicated. HIV-1_IIIB_ gp120 is T-tropic and works via the CXCR4 receptor most importantly was found to affect neurons (Bachis et al., 2006, 2009). Each vial of gp120 recombinant protein contains approximately 50 μg of phosphate-buffered saline (PBS) at a concentration of 1 mg/ml. Upon receiving the vial, we add 450 μl of PBS and aliquot the quantity into 50 tubes (10 μl/tube or 100 ng/μl) where it will be frozen and stored. 1 μl of protein will be added to each ml of media depending on the size of the plate.

### Chemical Reagents

AMD3100 (gp120-CXCR4 inhibitor) (Donzella et al., 1998) was kindly received from NIH AIDS Research Program and used at a concentration of 1 μM. Cells were treated with rolipram (30 μM, purchased from Sigma-Aldrich – 61413-54-5) or with salidroside (5 μM, purchased from Cayman Chemicals–10338-51-9) for 1 and 24 h, respectively before the addition of gp120 protein. Both chemicals are activators of cAMP.

### TUNEL Assay for *in situ* Apoptosis Detection

Apoptotic DNA degradation was detected by the TUNEL assay (30063 CF™488A TUNEL Assay–Biotium), according to the manufacturer’s instructions. The cells were cultured in 12-well plates. After treatment with gp120 for 24 h, cells were washed with PBS, fixed in 4% paraformaldehyde solution for 30 min at 4°C in the dark. Following washing with PBS, the cells were incubated in a permeabilization solution containing 0.2% Triton X-100 for 30 min at room temperature. Then DNA was labeled by incubating the cells with TUNEL reaction mix (Tdt enzyme and fluorescein-conjugated dUTP) in a humidified incubator for 60 min at 37°C in the dark. Finally, the percentage of TUNEL-positive cells was determined by flow cytometry. For positive control, a sample of fixed cells was incubated with 2 μg/mL of DNase I (Catalog # 17-141h) in PBS containing Ca^2+^ and Mg^2+^ or Reaction Buffer, for 60 min at 37°C. DNase I cleaves the DNA to generate a substrate for the end-labeling reaction. The DNase I reaction is stopped by washing for 15 min with PBS. For negative control samples, TUNEL reaction buffer is added without TdT Enzyme.

### Western Blot Assay

The whole-cell lysate was prepared using Radioimmunoprecipitation assay (RIPA) lysis buffer (25 mM Tris-HCl pH 7.6, 150 mM NaCl, 1% Triton, and 0.1% SDS) + protease and phosphatase inhibitor cocktail. 25 μg of the sample were loaded in each well. Protein concentrations were estimated using a Bradford Assay (Bio-Rad Catalog # 500-0006). Lysates were mixed with 6X loading dye containing β-ME followed by boiling at 95°C on a dry bath. The gel was then transferred to a nitrocellulose membrane. The membrane was blocked with 5% BSA for 1 h at RT. Primary antibodies were prepared in a 5% BSA solution as well and the membranes were incubated overnight at 4°C with gentle shaking. Antibodies used to detect the target proteins: PSD-95 (Santa Cruz [7E3]: sc-32290, monoclonal 1/100 dilution), CREB (Cell Signaling [D76D11] rabbit mAb #4820, 1/200 dilution, and [86B10] mouse mAb #9104, 1/500 dilution), pCREB^S133^ (Cell Signaling [87G3]: rabbit mAb #9198, 1:500 dilution), BDNF (Santa Cruz [N20]: sc-546, rabbit 1/100 dilution {*discontinued*}; and [5H8]: sc-65514, mouse 1/200), PGC1α (Santa Cruz [4A8]: sc-517380, mouse 1/100 dilution), KIF1B (Santa Cruz [H-190]: sc-28540, rabbit 1/100 dilution), Dyenin (Abcam [74.1]: ab23905, mouse 1/200 dilution), MIRO/Rho1 (Abcam mouse mAb #211363, 1/250 dilution), GAPDH (Abcam [6C5]: ab8245, mouse 1/500 dilution), E2F3 (Abcam rabbit #ab152126, 1/1000 dilution), PDE4 (Abcam rabbit #ab14628). Species-specific secondary antibodies were used from Santa Cruz and the membranes were incubated for 1 h at room temperature. Chemo luminescence was used to detect the band signal. The densitometry ratio of the bands was determined using an ImageJ that was normalized to the GAPDH.

### RNA Extraction

Total RNA was extracted from the sample using the SurePrep™ TrueTotal™ RNA purification kit from Fisher Bioreagents (BP280050). Nanodrop was used to determine the purity and concentration of the RNA extracted.

### MicroRNA

For microRNA expression cDNA was synthesized using miRCURY LNA Universal RT microRNA PCR from EXIQON (203301) with 100 ng of RNA. Primers for miR-34a and miR-134 were purchased from Exiqon. Results were expressed in relative gene level as compared to the untreated control. U6 was used as an internal control.

### qPCR Assay

cDNA was synthesized using SuperScript VILO cDNA synthesis kit (Invitrogen # 11754-050). The following primers were purchased from IDT: BDNF: (F)- 5′-gagcagctgc cttgatggttactt-3′; (R)- 5′-aagccaccttgtcctcggatgttt-3′. CREB: (F)- 5′-ggcagacagttcaagtccatg-3′; (R)- 5′-cgctttgggaatcagtt acac-3′. PGC1α: (F)- 5′-ccaaaccaacaactttatctc-3′; (R)- 5′-cacttaaggtgcgtt caatag-3′. PSD95: (F)- 5′-ggtggcagagcagagagatta-3′; (R)- 5′-tggctgagaagcactctgtg-3′. GAPDH: (F)- 5′-gccttccgtgttcctacc -3′; (R)- 5′-cctcagtgtagcccaagatg-3′. Results are expressed in relative gene expression level as compared to the untreated control. GAPDH was used as an internal control.

### Immunohistochemistry

#### gp120 Transgenic Mice

Imaging of gp120-tg mouse brains was done in Dr. Kaul’s lab (Sanford Burnham Presbys Medical Discovery Institute). WT and gp120-tg mice (express gp120 under GFAP promoter) were kindly provided by Dr. Lennart Mucke (Gladstone Institute) (Toggas et al., 1994) and maintained at Sanford Burnham Presbys Medical Discovery Institute. The mice (WT and gp120-tg), 9 months of age, were anesthetized with Isoflurane and transcardially perfused with 0.9% saline. The brains were quickly removed and fixed with 4% paraformaldehyde for 48 h at 4°C. The brain sections, 30 μm in thickness, were obtained for the histological studies. The slides were permeabilized with 1% Triton X-100 for 30 min followed by blocking with 10% heat/inactivated goat serum in PBS containing 0.5% Tween 20 for 1.5 h. The sections were then stained with CREB (Cell Signaling # 9197S) and MAP-2 (Sigma # MAB3418) overnight followed by Alexa Flour 488-labeled donkey anti-rabbit (Molecular Probes # A21206). Nuclear DNA was labeled with H33342. Per animal, at least three sagittal sections were analyzed, and each section’s five fields were recorded using Zeiss inverted Axiovert 100 M fluorescence microscope (Maung et al., 2014). All experiments involving gp120-tg mice were performed following NIH guidelines and approved by the IACUC of Sanford Burnham Presbys Medical Discovery Institute.

#### Tissue Preparation, Histology, Immunohistochemistry Cyclic AMP Response Element-Binding Protein

Mouse tissues were collected and fixed in 10% phosphate-buffered formaldehyde (formalin # HT5012, Sigma-Aldrich) for 24-48 h, dehydrated, and embedded in paraffin. Hematoxylin and eosin-stained sections were used for morphological evaluation purposes and unstained sections for IHC studies. Immunohistochemical staining was carried out according to standard methods. Briefly, 5 μm formalin-fixed, paraffin-embedded sections were deparaffinized and hydrated. Sections were then subjected to heat-induced epitope retrieval with Citrate buffer (pH 6.0). Endogenous peroxidases were quenched by the immersion of slides in a 3% hydrogen peroxide solution. The sections were incubated overnight with anti-phospho-CREB (Ser133) (87G3) Rabbit mAb (Rabbit, 1:200, 9198, Cell signaling) at 4°C in a humidified slide chamber. Immunodetection was performed using the Dako Envision+ polymer system and immunostaining was visualized with the chromogen 3, 3’-diaminobenzidine. The sections were then washed, counterstained with hematoxylin, dehydrated with ethanol series, cleared in xylene, and mounted.

### mtDNA Copy Number

Total genomic DNA was extracted from differentiated and gp120 treated SH-SY5Y cells with different treatment conditions. To measure mtDNA quantitative real-time PCR was performed with FastStart Universal SYBR Green Master (ROCHE # 04913914001). The primer sequences used for mtDNA were: mt (F)- 5′-cgaaaggacaagagaaataagg-3′ and mt (R)- 5′-ctgtaaagttttaagttttatgcg-3′. β-Globin (F)- 5′-caa cttcatccacgttcacc-3′ and (R)- 5′-gaagagccaaggacaggtac-3′. The primers were purchased from IDT. Note that mtDNA was measured using qPCR while the copy number was measured as a ratio of an mt gene from the D loop over a nuclear gene (β globin).

### Mitochondrial Mobility (Kymograph)

SH-SY5Y cells were seeded on the glass bottom plates and transfected with 0.5 μg of Mito dsRED plasmid (Clontech # 632421) using lipofectamine 2000 (Invitrogen # 11668027). The transfection media (Opti-MEM # 31985-062) was replaced with the differentiation media after 4–6 h and the cells were left to differentiate for 96 h and then treated with 100 ng/ml of gp120 protein for 24 h and/or 30 μM of rolipram for 1 h. The cells were visualized using Leica confocal microscope Leica (DMI4000) for live-cell imaging. A heated 37°C temperature-controlling chamber filled with 5% CO_2_ surrounding the microscope stage was used to keep the cells alive. Images were captured every 5 s for a total of 5 min. The area of the cell visualized was focused on the processes and not the cell body. Mitochondrial movement along the processes was represented in the form of a kymograph by using Image J. Mitochondria moving at a velocity higher than 0.1 μm/s were considered mobile. At least thirty axons were recorded. The vertical straight line represents the immobile mitochondria whereas, the dotted line along the horizontal axis represents the mobile mitochondria.

### ATP Assay

ATP was measured with a luciferin – luciferase bioluminescence assay using an ATP determination kit from Molecular Probes (#A22066) as per the manufacturer’s protocol. Briefly, the standard curve was measured using different concentrations of ATP solution (1 nM-1 μM) with the standard solution. The cells were subjected to a freeze and thaw cycle (3x) to release the ATP. The cells were centrifuged at 12000rpm for 10 min. 10 μl of supernatant was used for every 100 μl of the standard solution. The spectrophotometer was used to measure the luminescence. The data is expressed as a relative ATP level as compared to the untreated control (Mock). (Luciferin + ATP + O_2_ (Mg^2+^)(Luciferase) → oxyluciferin + AMP + pyrophosphate + CO_2_ + light).

### ADP/ATP Ratio

Changes in the ADP/ATP ratio were measured using the ADP/ATP ratio assay kit (MAK135) purchased from Sigma-Aldrich which is based on a luciferin–luciferase assay. The assay involved two steps: (i) ATP reacts with the substrate D luciferin and produces light in the presence of luciferase–measuring the intracellular ATP concentration; (ii) ADP is converted to ATP through an enzymatic reaction, followed by the reaction of ATP with D-luciferin. The second light intensity measurement represents the total ADP and ATP concentration in the sample. The ATP reagents were prepared where 90 μl/well were added to the samples and incubated for 1 min at room temperature followed by a luminescence reading. This operation was repeated with 10 min of incubation. Immediately after the second reading, 5 μl of ADP reagent was added to each well, incubated for 1 min at RT, and the luminescence was read. The ADP/ATP ratio was then calculated.

### NAD^+^/NADH Ratio

Differentiated and gp120-treated SH-SY5Y cells were washed with cold PBS and centrifuged at 2,000 rpm for 5 min. The cells were homogenized with 400 μl of NADH/NAD buffer, vortexed for 10 s, and then centrifuged at 13,000 × *g* for 10 min to remove any insoluble materials. The extracted NAD/NADH supernatants were then transferred into a 96-well plate with a final volume of 50 μl to measure NAD^+^. The second set was prepared by aliquoting 200 μl of the samples into microcentrifuge tubes and heating them to 60°C for 30 min. The samples were then cooled on ice, spun, and transferred to the plate. This is used to measure NADH. 100 μl of the Master Reaction and 10 μl of the NADH Developer were added to each sample. The absorbance was measured at 450 nm at different time points (30, 60, and 120 min) using a Modulus microplate reader. The ratio of NAD^+^/NADH was calculated using the following formula:


$$\begin{array}{c} \text{Ratio}=\left({\mathrm{NAD}}_{\text{total}}-\mathrm{NADH}\right)/\mathrm{NADH}({\mathrm{NAD}}_{\text{total}}\\ \;=\mathrm{Total}\mathrm{amount}\mathrm{of}\mathrm{NAD}\left[\mathrm{NAD}+\mathrm{NADH}\right];\mathrm{NADH}\\ \;=\mathrm{Amount}\mathrm{of}\mathrm{NADH}) \end{array}$$

### Proteomics Assay

SH-SY5Y cells were treated in duplicate with gp120B and gp120C for 48 h. The cells were collected and processed for proteomics analysis. Briefly, for label-free global proteomics studies, the proteins were extracted by adding 6M guanidium hydrochloride buffer and dilution buffer (25 mM Tris, 10% acetonitrile). They were then digested with Lys-C for 4 h at 37 ^0^C. Second digestion was achieved by overnight incubation with trypsin. The incubated solution was acidified and centrifuged at 4,500 × *g* for 5 min. The supernatants consisting of peptides were loaded onto activated in-house-made cation stage tips (Zhang et al., 2018; Molina-Franky et al., 2021). The peptides from each sample were eluted into six fractions using elution buffers as previously described (McBrearty et al., 2021). Mass spec analyses were performed on these fractions using the ELITE mass spectrometer (Thermo Fisher Scientific). The desalted tryptic peptide samples were loaded onto an Acclaim PepMap 100 pre-column (75 μm × 2cm, Thermo Fisher Scientific) and separated by an Easy-Spray PepMap RSLC C18 column with an emitter (2 μm particle size, 15 cm × 50 μm ID, Thermo Fisher Scientific) by an Easy nLC system with Easy Spray Source (Thermo Fisher Scientific). To elute the peptides, a mobile-phase gradient is run using an increasing concentration of acetonitrile. Peptides were loaded in buffer A (0.1% (v/v) formic acid) and eluted with a nonlinear 145-min gradient as follows: 0–25% buffer B (15% (v/v) of 0.1% formic acid and 85% (v/v) of acetonitrile) for 80 min, 25–40% B for 20 min, 40–60% B for 20 min and 60–100% B for 10 min. The column was then washed with 100% buffer B for 5 min, 50% buffer B for 5 min, and re-equilibrated with buffer A for 5 min. The flow rate was maintained at 300 nl/min. Electron spray ionization was delivered at a spray voltage of -2000 V. MS/MS fragmentation was performed on the five most abundant ions in each spectrum using collision-induced dissociation with dynamic exclusion (excluded for 10.0 seconds after one spectrum), with automatic switching between MS and MS/MS modes. The complete system was entirely controlled by Xcalibur software. Mass spectra processing was performed with Proteome Discoverer v2.4. The generated de-isotoped peak list was submitted to an in-house Mascot server 2.2.07 for searching against the Swiss-Prot database (Release 2013_01, version 56.6, 538849 sequences) and Sequest HT database. Both Mascot and Sequest search parameters were set as follows: species, homo sapiens; enzyme, trypsin with maximal two missed cleavage; fixed modification, cysteine carboxymethylation; 10 ppm mass tolerance for precursor peptide ions; 0.02 Da tolerance for MS/MS fragment ions.

### Synaptophysin Vesicles Number and Neurite Retraction and Distribution Assays

CellLight Synaptophysin RFP BacMac 2.0 from Life Technology (catalog# C10610) was used to transduce differentiated primary mice neurons or SH-SY5Y cells at MOI of 5 for 24 h following the manufacturer’s protocol. Images were taken using the EVOS*-fl* fluorescence microscope (Thermo Fisher Scientific) and Image J was used to count the number of synaptophysin vesicles or to measure neurite retraction.

### Transmission Electron Microscopy

Differentiated SH-SY5Y cells were grown on 100 × 20 mm tissue culture-treated dishes (Celltreat ^®^ Scientific Products # 229620) for 3 days in F-12/DMEM (50/50) supplemented with sodium pyruvate, nonessential amino acids, and 10% FBS. Cells were then treated with 100 ng/ml gp120 recombinant Protein or PBS for 24 h. The cells were then collected, centrifuged, and fixed. To fix the cell, 500 μl of formaldehyde glutaraldehyde, 2.5% in 0.1M Sod. Cac. buffer, pH 7.4 (Electron Microscopy Sciences # 11650) was added slowly to the top of the pellet. The samples were then put on ice and transferred to the EM facility. After fixation cells were centrifuged at 1000 × *g*, washed 3 times with 0.1M Cacodylate buffer. The cell pellet (∼ 50 μl) was mixed with 100 μl of 5% agarose and centrifuged at 1000 × *g* for 10 min. Tubes with cell pellets were transferred at 4°C/ice for 1 h to solidify agarose. The cell pellets in agarose were postfixed with 1% OsO_4_ in the solution for 1 h at room temperature and washed 3 times with DD H_2_O. Samples were further processed for dehydration with serial changes of Ethanol, were embedded into Epon 812 resin (Electron Microscope Sciences, United States), and polymerized at 65°C for 72 h. Embedded samples were cut thin (80–100 nm) using Leica UC6 microtome, collected on 200 mesh copper grids (Electron Microscopy Sciences, USA), and stained with 2% Uranyl Acetate for 12 min at RT followed by staining with Reynolds lead citrate for 6 min at RT. Samples were visualized with FEI Tecnai T12 transmission electron microscope, at 100 kV, equipped with 2K × 2K Megaplus camera Model ES 4.0 (Roper Scientific MASD, San Diego, CA, United States). Mitochondria are highlighted in blue and numbered before using image J to measure area and perimeter. Any structure that cannot be identified as a mitochondrion (i.e., no evidence of cristae or double membrane) or that has been cut off when acquiring the image was not included. Standard error was used (*p* = 0.378 [*t*-test]).

### Stereotaxic Surgery and Spatial Memory Testing

#### Stereotaxic Surgery

Mice were singly housed with *ad-libitum* access to food and water before and after surgery. 8–10 weeks old C57B1/6J male mice were anesthetized with isoflurane and received bilateral stereotaxic injections (1 μl volume per injection site) of either saline or gp120 (125 ng/μl) into the hippocampi at rostral (–1.7 mm A/P, 1.2 mm M/L, 2 mm D/V from bregma) and caudal (–2.7 mm A/P, 2 mm M/L, 2.1 mm D/V from bregma) coordinates for a total of 4 intrahippocampal injections per mouse. Mice then received intraperitoneal injections of saline or rolipram (1 mg/kg) immediately after surgery and 36 h after surgery. 72 h post stereotaxic injection, spatial memory was tested using the object location memory test. All procedures were approved by the Baylor College of Medicine Institutional Animal Care and Use Committee.

#### Object Location Memory Testing

The object location memory test requires that mice learn and remember the positions of two objects in an arena. Extra-arena spatial cues exist to orient the mice during the training and testing phases. For training, two identical flasks were placed at adjacent far corners of the arena, and animals were allowed to explore both flasks in 3 trials of 3 min each with 3 min intervals. The time mice spent exploring each flask was recorded by the experimenter. After a delay of 24 h, mice were returned to the arena for the test phase. In this phase, one flask was displaced to the adjacent empty corner. Mice were given 3 min to explore both flasks, and the number of times spent exploring each flask was recorded. An increase in time spent with the displaced object during the test phase relative to the training phase is indicative of intact spatial memory.

### Statistical Analysis

All the experiments were repeated at least in triplicate. Statistical analysis was performed using a one-way analysis of variance with a *post hoc* Student’s *t*-test. Data are expressed as the mean of ±S.D. Results were judged statistically significant if *p* < 0.05 by analysis of variance. (Marked in the figure as *p < 0.05; ^**^p < 0.01; ^***^p < 0.001 where needed). Data were plotted either using GraphPad Prism version 5.0 or 7.0.

### Human and Animal Statement

All experiments involving gp120-tg mice were performed *per* NIH guidelines and approved by the IACUC of Sanford Burnham Presbys Medical Discovery Institute. Similarly, all animal procedures were approved by the Baylor College of Medicine Institutional Animal Care and Use Committee.

## Results

### HIV-1 gp120 Alters PGC1α Expression

**FIGURE 1:**
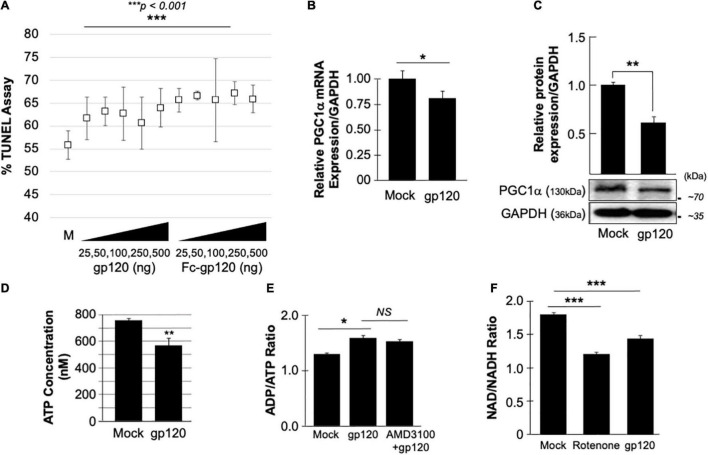
gp120 promotes the loss of mitochondrial energy. **(A)** *gp120 concentration and cell viability.* Differentiated SH-SY5Y cells were treated with an increasing concentration of gp120 proteins for 24 h then subjected to TUNEL assay as indicated. The results are statistically significant using the Student’s *t*-test. The data shown are from a single experiment that was replicated 3 times and was statistically significant. **(B,C)** Expression levels of PGC1α mRNA **(B)** and protein **(C)** isolated from untreated or gp120-treated SH-SY5Y as obtained by qPCR and western blot assays, respectively. Quantitative analysis of the western blot was presented along with the bands as a bar graph. The data shown are from a single experiment that was replicated 3 times and was statistically significant. **(D)** The concentration of ATP produced by SH-SY5Y cells treated with gp120 compared to mock cells. **(E)** Measurement of ADP/ATP ratio in SY-SY5Y cells untreated or treated with gp120 or AMD3100/gp120. AMD3100 (1 μM) (inhibitor of CXCR4) pre-treatment was used as a control to inhibit gp120-CXCR4 association. **(F)** Measurement of NAD/NADH ratio in mock, gp120-treated, and rotenone-treated cells. Rotenone is a complex I inhibitor and was used as a positive control. The experiments were done in triplicate and the *p*-values were calculated using the Student *t*-test (Mean ± S.D.). *p < 0.05; **p < 0.01; ***p < 0.001.

The neuroblastoma cell line, SH-SY5Y, was used to assess the concentration of gp120 necessary to affect the cell function without hindering cell viability severely. SH-SY5Y cells were differentiated with retinoic acid for 72 h and then treated with increasing concentrations of recombinant gp120 proteins {HIV_IIIB_ gp120, or HIV-_JR–CSF_-Fc-gp120 (Binley et al., 2006), NIH-AIDS Reagents Program} for 24 h. TUNEL assay was performed and showed that 15–20% of cells contain fragmented DNA (green fluorescence) (which could also indicate dead cells) (Figure 1A). Based on these results, we chose to use 100 ng/ml of HIV-1 gp120 protein in our experiments.

The effect of gp120 on mitochondrial deregulation has been shown (Avdoshina et al., 2016; Fields et al., 2016a,b; Rozzi et al., 2017; Fields and Ellis, 2019), however, the mechanisms involved remain to be elucidated. We started by assessing the role of gp120 on PGC1α, a protein responsible for mitochondrial biogenesis. Differentiated SH-SY5Y cells were treated with 100 ng/ml of HIV_IIIB_ gp120 for 48 h. The cells were then divided into two groups where the mRNA was isolated from one group and subjected to qPCR (Figure 1B) while protein extracts were isolated from the second group and processed for Western blot (Figure 1C). The addition of gp120 protein led to decreased mRNA and protein expression of PGC1α. Change in PGC1α protein expression was measured and presented as a histogram in Figure 1B. These data suggest that a loss of PGC1α expression causes deleterious effects on mitochondrial biogenesis.

### HIV-Gp120 Deregulates Mitochondrial Energy

The reduction of PGC1α protein expression sensitizes the cells to oxidative stress, resulting in ROS accumulation and a decrease in ATP generation (Rius-Pérez et al., 2020). To validate this hypothesis, SH-SY5Y cells were treated with 100 ng/ml of recombinant HIV_IIIB_ gp120 for 24 h after which the cells were collected, and ATP levels were measured (Figure 1D). The addition of gp120 decreased the ATP concentration compared to mock cells. The ADP/ATP ratio was also measured in the presence of HIV_IIIB_ gp120 protein or gp120 and AMD3100. AMD3100 pre-treatment was used as an inhibitor for CXCR4 to inhibit the effect of gp120. As expected, the ratio of ADP/ATP increased in gp120-treated cells even when AMD3100 was used (Figure 1E). These results corroborate the literature regarding the ability of gp120 to enter the cells using different routes like pinocytosis (Berth et al., 2015).

Nicotinamide adenine dinucleotide (NAD) is used as a marker for aging and may help to explain the ATP reduction and ROS production seen in these cells. The addition of recombinant HIV_IIIB_ gp120 to SH-SY5Y cells lowered the NAD^+^/NADH ratio (Figure 1F). Rotenone was used as a positive control because it works by interfering with the electron transport chain in mitochondria (Goldstein et al., 2015).

### HIV-1 gp120 Causes a Loss of Mitochondrial Cristae and Alters Mitochondrial Ultrastructure

**FIGURE 2:**
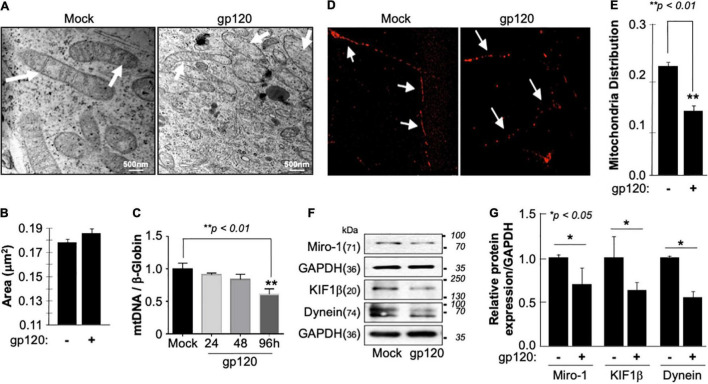
Deregulation of mitochondrial ultrastructure, mtDNA, and distribution in the presence of HIV-1 gp120. **(A)** Differentiated SH-SY5Y cells were treated with either PBS or recombinant HIV-1 gp120 protein at 100 ng/ml for 24 h before being fixed and processed for electron microscopy. Mock cells show mitochondria with intact cristae (arrows) while HIV-1 gp120-treated cells show fewer and shorter cristae as well as large open spaces (swelling mitochondria) where no visible cristae are observed. **(B)** Mitochondrial areas were measured (nm^2^) using ImageJ. Any structure that cannot be identified as a mitochondrion (i.e., no evidence of cristae or double membrane) or that has been cut off when acquiring the image was not included. Standard error was used. **(C)** mtDNA copy number was determined by taking the mtDNA/β-Globin ratio. Fold change in mtDNA copy number in HIV-1 gp120-treated primary human neurons relative to the mock is shown. The bar graph represents mean values ± S.E.M. *p* < 0.001 (*n* = 4). **(D)** A representation of the mitochondria distribution, and density in the neuronal processes of the differentiated SH-SY5Y cells. Images were taken after transfecting the cells with 0.5 μg of Mito dsRED plasmid before differentiation. The images were taken post 24 h of the respective treatment using a confocal microscope. Arrows represent the gaps between the mitochondria. The data shown are from a single experiment that was replicated 3 times using different cell batches. **(E)** Ratio of moving mitochondria was measured and represented as a scatter plot using a one-way analysis of variance with a post hoc Student’s t-test. Statistical significance level, *p* < 0.01. **(F)** Expression of motor and mitochondrial proteins (Miro-1, KIF1β, and Dynein) isolated from untreated or HIV-1 gp120-treated SH-SY5Y as obtained by western blot analysis. **(G)** Quantitative analysis of the western blot presented along with the bands as a bar graph using the Student’s *t*-test statistical significance level, *p* < 0.05 or 0.01.

The inner membrane of the mitochondria, also known as mitochondrial cristae due to their wavy shape, is the site of electron exchange and ATP production. Loss of the cristae leads to dissociation of ATP synthase dimers and impaired ability of the mitochondria to supply the cell with enough ATP required for cell function (Pathak et al., 2013). Therefore, to determine if the lower level of ATP observed is due to the loss of cristae, we treated SH-SY5Y cells with 100 ng/ml of recombinant HIV_IIIB_ gp120 protein for 24 h and visualized them using an electron microscope. The ultrastructure of the mitochondria, including the cristae, was examined and compared to that of control (mock) cells (Figure 2A). The mitochondria shape changed, and the cristae were lost in gp120-treated cells. This ultrastructural alteration could explain the decrease in ATP production and increased ROS seen in these cells. No significant changes in the mitochondrial area were observed (Figure 2B).

Low ATP level is also associated with decreased mitochondrial DNA (mtDNA) copy number. Therefore, the mtDNA copy number was measured in human primary cultures of neurons (hippocampal) (Figure 2C). The addition of HIV_IIIB_ gp120 for 24, 48, and 96 h led to a reduction of mtDNA copy numbers, especially during 96 h. Note that a 20% change in the mtDNA copy number is considered significant (Kim et al., 2019).

Next, we examined the mitochondrial distribution and density in SH-SY5Y cells treated with 100 ng/ml of recombinant HIV_IIIB_ gp120 protein. These cells also expressed a Mito dsRed plasmid to visualize the mitochondria. Mitochondria distribution and density were reduced in gp120-treated cells compared to the mock untreated as obtained by confocal microscopy (Figures 2D,E).

Additionally, we tested the expression level of Miro-1 (aka Rho T1), KIF1β, and Dynein proteins. Miro is a protein that links mitochondria to KIF1β and Dynein motor proteins, allowing the mitochondria to move along the microtubule (Nangaku et al., 1994; Hollenbeck and Saxton, 2005). The addition of HIV_IIIB_ gp120 resulted in a decrease in Miro-1, KIF1β, and Dynein expression levels (Figure 2F). These results confirm the ability of gp120 to cause mitochondrial movement impairments. Changes in protein expression levels were quantified using Image J and presented in Figure 2G. These results corroborate previous data regarding the ability of gp120 to cause mitochondrial damage.

### HIV-Gp120 Cause Synaptic Plasticity Deregulation

**FIGURE 3:**
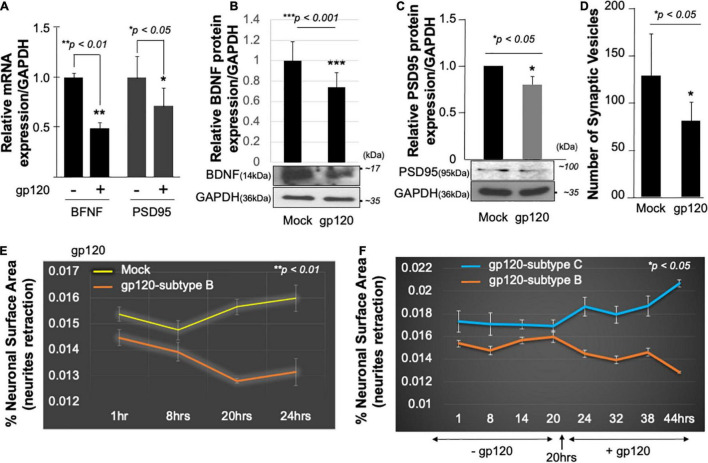
HIV-1 gp120 alters synaptic plasticity. Expression of BDNF and PSD95 mRNA prepared from untreated or HIV-1 gp120-treated SH-SY5Y for 24 as obtained by qPCR **(A)** and Western blot analysis using an anti-BDNF antibody **(B)**, anti-PSD95 **(C)**, and anti-GAPDH antibodies **(B,C)**. Quantitative analysis of the western blot is presented on the top of the bands as a bar graph. Student’s *t*-test statistical significance level, *p* < 0.001. BDNF and PSD95 data are from a single experiment that was replicated 2 times. **(D)** *Quantification of synaptophysin-positive synaptic vesicles–* Synaptophysin was expressed using Cell Light Transduction at MOI of 5. HIV-1 gp120 protein or mock treatment was added to primary mouse neurons 24 h after the transduction. Synaptophysin vesicles were counted using ImageJ for each condition. Images from at least 10 different fields were taken for statistical analysis *p* < 0.05. Student’s *t*-test statistical significance level, *p* < 0.05. **(E,F)** SH-SY5Y cells were treated with 100 ng/ml of HIV-1 gp120 (subtype B) **(E)**. Live-cell images and bright-field contract images were acquired every 30 min from the same field for the indicated times. The images were analyzed with ImageJ software and the surface area covered by the cells was normalized against the total number of the cells at each time point. The experiment was repeated with 100 ng/ml of gp120 (clades B and C) **(F)**. Supplemented medium added at 20 h. The experiments were repeated 2 times and the images from at least 10 different fields were taken for each set for statistical significance (*p* < 0.05).

Mechanistically, functional PGC1α relies on several transcription factors such as the brain-derived neurotrophic factor (BDNF) and its ability to promote the phosphorylation of CREB, which in turn activates the PGC1α promoter (Cheng et al., 2012). Hence, we examined the expression level of BDNF protein in the presence of gp120. SH-SY5Y cells were treated with 100 ng/ml of recombinant HIV_IIIB_ gp120 for 24 h after which the cells were collected and processed for qPCR and Western blot (Figures 3A,B). The addition of gp120 protein resulted in a decrease in BDNF mRNA and protein expression levels compared to the mock untreated. Changes in BDNF protein expression levels were measured using Image J (Figure 3B, histogram). These results corroborate previous data regarding the decrease of BDNF in HIV-human brain tissues (Michael et al., 2020).

It has been shown that the mitochondria and BDNF play a significant role in controlling fundamental processes in neuroplasticity and have been linked to long-term potentiation and PSD-95 regulation (Markham et al., 2014; Sen, 2019). Therefore, we determined the effect of BDNF and PGC1α loss on two synaptic markers, synaptophysin (a protein involved in synaptic transmission) and PSD-95 (is a scaffold protein present on the dendrites of the postsynaptic neurons and has been used as a synaptic marker, and stabilization of synaptic changes during long term potentiation) (Zhang and Lisman, 2012).

Following the same procedure as for BDNF, SH-SY5Y cells were cultured and treated with 100 ng/ml of recombinant HIV_IIIB_ gp120 for 24 h after which the cells were collected and processed for qPCR and Western blot. The addition of gp120 protein resulted in a decrease in PSD95 mRNA (Figure 3A), and protein expression levels compared to the mock untreated using anti-PSD-95 or -GAPDH antibodies (Figure 3C).

Next, primary human neurons (E18) were used and the synaptophysin vesicles were labeled with red fluorescence for 22 h before adding 100 ng/ml of recombinant HIV_IIIB_ gp120 protein for an additional 24 h. Images were acquired on live cells 24 h post gp120 treatment using the EVOS-*fl* microscope. We observed a decrease in the number of synaptophysin vesicles in gp120-treated cells compared to the mock untreated cells (Figure 3D).

Low expression of PSD-95 protein points to synapse shortening and loss of synaptic plasticity. We validated this observation by measuring the neuronal surface area in live cells. The shortening in neurite length was observed in SH-SY5Y cells treated with HIV_IIIB_ gp120 compared to mock cells (Figure 3E).

We repeated this last experiment using 100 ng/ml of recombinant gp120 proteins prepared from HIV-1 (clades B or C). HIV-1 gp120 (subtype C) was shown to be responsible for decreased neurovirulence of clade C (Rao et al., 2014). We found that the addition of HIV_IIIB_ gp120-B but not gp120-C causes shortening of neurites length in SH-SY5Y (Figure 3F). These results suggest that HIV-1 gp120 can cause neurite retraction leading to altered spatial memory.

### HIV-1 Gp120-Induced Mitochondrial Dysfunction Is Cyclic AMP Response Element-Binding-Dependent

Among its many functions, CREB protein has been shown to modulate mitochondrial biogenesis, directly and indirectly, through regulating the transcription of several genes such as *pgc1α* (directly) and *bdnf* (indirectly) (Cheng et al., 2010; Kang et al., 2017). Therefore, we sought to examine the effect of gp120 on CREB function and whether gp120 is using CREB protein to cause the loss of mitochondrial energy. Since CREB function depends on its phosphorylation on serine residue 133 (pCREB^S133^), we also examined its expression levels in the presence of gp120 protein.

**FIGURE 4:**
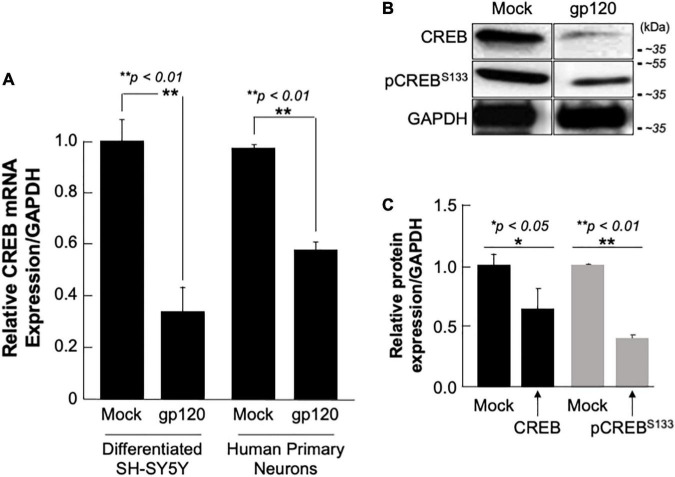
Deregulation of CREB in HIV-1 gp120 treated cells. **(A)** Expression of CREB mRNA isolated from differentiated SH-SY5Y and human primary neurons from a 26-week-old fetal brain. These were then treated with 100 ng/ml of HIV-1 gp120 IIIB for 24 h and CREB mRNA expression was measured by qPCR (repeated 3x) and normalized to GAPDH as an internal control. Data represent the mean ± S.D. Results were judged statistically significant if *p* < 0.05 by one-way analysis of variance and ANOVA test. (*p < 0.05 and **p < 0.01) (M = Mock, GP = gp120). **(B)** Expression of CREB protein using 50 μg of extracts isolated from untreated or HIV-1 gp120-treated SH-SY5Y as obtained by Western blot using anti-CREB, -pCREB^S133^, or -GAPDH (used as a protein loading control) antibodies. **(C)** Quantification of the relative protein level was determined from the band intensity using ImageJ software and normalized relative to the GAPDH. Bar graphs represent the means ± SD of at least two independent experiments.

SH-SY5Y and primary human neurons were treated with 100 ng/ml of recombinant gp120 IIIB protein for 24 h. mRNA and protein expression levels were then measured by qPCR. The expression of CREB mRNA is significantly lower in cells treated with HIV_IIIB_ gp120 (GP) compared to mock untreated (M) (Figure 4A). CREB protein (total and phosphorylated) expression levels were also assessed by Western blot and show decreased expression in gp120-treated SH-SY5Y cells (Figure 4B). Changes in CREB expression levels were measured using Image J and presented in Figure 4C.

### Restoring Cyclic AMP Response Element-Binding Expression and Function in Rolipram-Treated Cells

**FIGURE 5:**
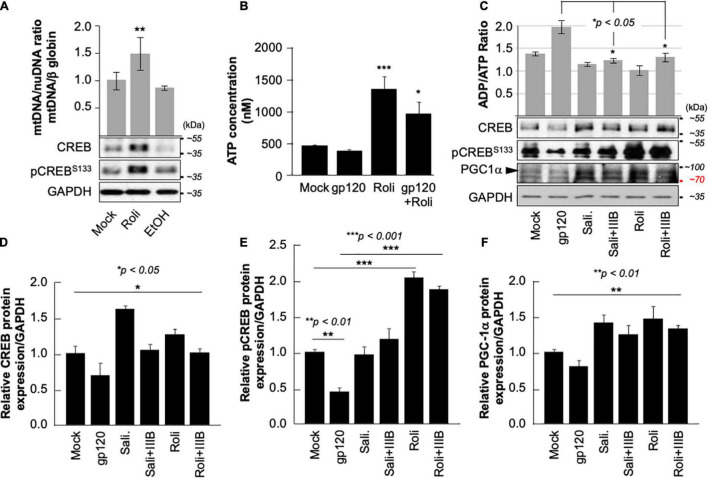
Rolipram restores CREB phosphorylation and targets. **(A)** HEK293 cells were treated with ethanol (200 mM for 24 h) to repress and prevent pCREB^S133^ function or with rolipram (30 μM/1 h) to induce pCREB^S133^ expression and function. mtDNA copy number is represented by measuring the ratio of mtDNA/β-Globin. Expression levels of total CREB and pCREB^S133^ were also evaluated in HEK293 extracts using western blot analysis. Fold change in CREB expression relative to GAPDH was also measured and deemed significant (*p* < 0.05). **(B)** Differentiated SH-SY5Y cells were treated with 30 μM of rolipram for 1 h before adding 100 ng/ml of HIV-1 gp120 protein for 24 h after which the reaction was stopped, and cells were collected. ATP levels in the nM range were measured through a luciferase assay (*n* = 2) **(C)**. Measurement of ADP/ATP ratio in mock, HIV-1 gp120-treated, rolipram (30 μM/1 h) ± HIV-1 gp120-treated, and salidroside (5 μM/24 h) ± HIV-1 gp120-treated SH-SY5Y cells as indicated. The ratio is presented as a scatter plot and changes are considered significant (*p* < 0.05) (*n* = 3). The lower panel represents the protein expression of total CREB, pCREB^S133^, and PGC1α in SH-SY5Y cells subjected to different treatment conditions as obtained by Western blot analysis. GAPDH is represented as the loading control. **(D–F)** Quantitative analysis of the western blots presented along with the bands. The experiments were done in triplicate and the *p*-values were calculated using the Student *t*-test (Mean ± S.D.). (*p < 0.05; **p < 0.01; ***p < 0.001).

Next, we aim to restore CREB expression and phosphorylation using rolipram. Rolipram (C_16_H_21_NO_3_) was developed as a potential antidepressant drug in the early 1990s (Egawa et al., 1997; Zhong et al., 2016). Many functions were associated with rolipram use in animals including restoring spatial memory through reactivation of CREB. Rolipram has since been discontinued due to its significant side effects. Ethanol (EtOH), a proven CREB inhibitor known to cause short memory lapse by dephosphorylating CREB protein and inhibiting its function (White et al., 2000; Liu et al., 2014) was used as a negative control. HEK-293 cells, cultured in duplicate, were treated with 200 mM of EtOH for 24 h or with 30 μM of rolipram for 1 h. The first set was subjected to Western blot using anti-CREB, -pCREB^S133^, and -GAPDH antibodies, while mitochondrial DNA (mtDNA) copy number was measured using the second set. As expected, treatment of the cells with EtOH decreased mtDNA copy number (Figure 5A- bar graph) and pCREB^S133^ expression level (Figure 5A, lower panel) while the addition of rolipram restored both (*the concentration and time of treatment of EtOH and Rolipram were chosen from publications using similar cells*) (Liu et al., 2014).

The ATP production was also measured in the presence of rolipram. Differentiated SH-SY5Y cells were treated with rolipram (30 μM) for 1 h and then treated with 100 ng/ml of HIV_IIIB_ gp120 for 24 h. A decrease in the ATP level was observed in gp120-treated cells but not in cells treated with rolipram or gp120 and rolipram (Figure 5B).

Similarly, gp120 failed to negatively affect the ratio of ADP/ATP in differentiated SH-SY5Y cells treated with rolipram (30 μM) for 1 h or with salidroside (5 μM) for 24 h (another CREB activator) (Jin et al., 2016) indicating more ATP production in the presence of these CREB activators (Figure 5C- bar graph).

Further, the addition of HIV_IIIB_ gp120 failed to decrease the protein expression levels of total CREB, pCREB^S133^, and PGC1α in the presence of rolipram or salidroside as shown by Western analysis (Figure 5C- lower panel). Changes in CREB, pCREB^S133^ and PGC1α protein expression levels were quantified using Image J and considered significant (Figures 5D–F).

Interestingly, all measurements of mitochondrial dysfunction including PGC1α caused by the addition of gp120 protein were reversed in the presence of rolipram or overexpressed CREB. These results indicate a rescue effect of treatment with CREB or CREB-activators.

**FIGURE 6:**
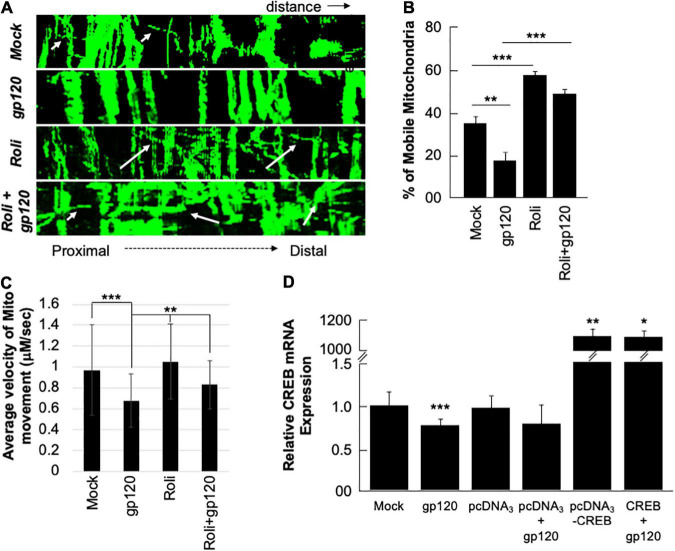
Rolipram and over-expressed CREB improve mitochondrial movement. **(A)** Kymographs representing mitochondrial movement in mock, HIV-1 gp120-treated (100 ng/ml), rolipram-treated, and rolipram + HIV-1 gp120-treated SH-SY5Y cells. The horizontal axis represents the distance, and the vertical axis represents the time. Mobile mitochondria are distinguished by the diagonal dotted lines moving along the horizontal axis (arrows) and stationary mitochondria are represented by vertical lines. The mitochondria were tagged using Mito ds-RED transfection and the movement was visualized using confocal microscopy. ImageJ was used to create a kymograph. Data is the representation of at least 15–20 neurites *per* treatment condition each repeated 3 times (*n* = 60–65 total neurites *per* treatment condition). The data shown are from a single experiment that was replicated 2 times. **(B)** Quantification of the percentage of mobile mitochondria. **(C)** The average velocity of the mobile mitochondria (*n* = 60-65 per treatment condition). Data represent the mean ± S.D. Results were judged statistically significant if *p* < 0.05 by analysis of variance. (*p < 0.05; **p < 0.01; ***p < 0.001). **(D)** Two sets of SH-SY5Y (1 × 10^5^) cells were transfected with 0.5 μg of pcDNA3 or pcDNA3-CREB expression plasmid as indicated. 24 h post-transfection the cells were differentiated and then treated with 100 ng/ml of gp120 protein for an additional 24 h. The expression level of CREB mRNA was measured.

Next, we sought to determine whether the mitochondria movement can be restored as well. SH-SY5Y cells were transfected with 0.5 μg of Mito dsRed plasmid for 24 h, differentiated, and then treated with rolipram (30 μM) for 1 h before adding 100 ng/ml of HIV_IIIB_ gp120 for 24 h. Moving mitochondria were observed using confocal microscopy (Figure 6A). Treatment with HIV-1 gp120 protein reduced the ratio of mobile mitochondria from 38% to 18% (Figure 6B). There was a significant difference in the ratio of moving mitochondria in neurons treated with rolipram (59%) and/or rolipram + gp120 (50%) compared to the untreated or gp120 treated cells excluding the possible false-positive effect brought by protein addition. The average velocity of mitochondrial movement is presented in Figure 6C.

To validate our data and the role of CREB as well the dependency of gp120 effect on functional CREB, we overexpressed CREB. Two sets of SH-SY5Y cells were transfected in triplicate with 0.5 μg of the empty vector (pcDNA_3_) or with the pcDNA_3_-CREB expression vector. Twenty hours later the cells were differentiated and then treated with 100 ng/ml of HIV_IIIB_ gp120 for an additional 24 h. We found that the expression of CREB mRNA is significantly lower in cells treated with gp120 or transfected with the empty vector and then treated with gp120 compared to controls. However, this expression increases dramatically in cells transfected with pcDNA_3_-CREB in the absence and presence of gp120 when compared to the Mock or cells transfected only with the empty vector alone (Figure 6D). These results confirmed our hypothesis that gp120 impairs spatial memory through CREB protein.

### Cyclic AMP Response Element-Binding Expression Decreases With Memory Impairment in Mice

**FIGURE 7:**
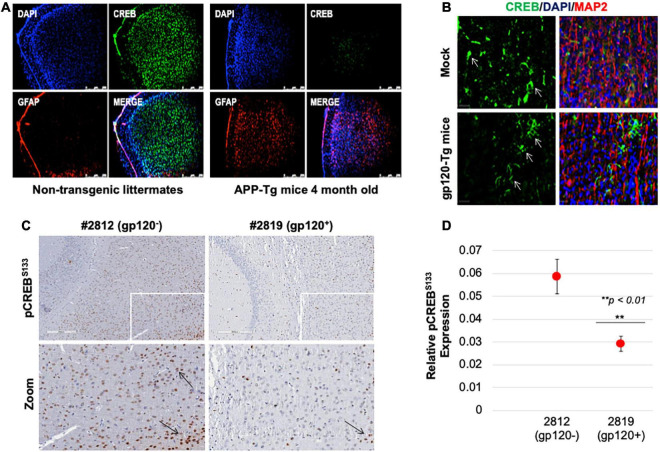
Cyclic AMP response element-binding expression level is altered in brain tissues. **(A)** Expression level of CREB protein (green) was observed in the brain tissues of 4-month-old APP-transgenic mice compared to non-transgenic littermates. Immunohistochemistry was performed using antibodies for CREB (green), and glial fibrillary acidic protein (GFAP) to visualize astrocytes (red). Nuclei were stained using DAPI (blue). **(B)** The expression of total CREB (green), and MAP2 (red) to visualize neurons, in hippocampal brain slides of 9-month-old HIV-1 gp120 transgenic mice (gp120-Tg) and age-matched control mice. Nuclei were stained using DAPI (blue). **(C)** The expression of pCREB^S133^ (brown spots) in the cortex area of 9-month-old HIV-1 gp120 transgenic mice (2819- gp120-Tg) and age-matched control mice (2812) (*n* = 2). **(D)** Quantification of the fluorescence intensity of pCREB^S133^ labeling. Data represent the mean ± S.D. Data shown in panels B and C are from a single experiment that was replicated 2 times each and was statistically significant. Results were judged statistically significant if *p* < 0.05 by analysis of variance. (**p < 0.01). Arrows are pointing at CREB protein.

Next, we sought to determine the rolipram effect *in vivo* using a mouse model. First, we examined CREB expression. Reduced CREB expression and loss of its phosphorylation have been observed in patients suffering from diseases like AD, PD, ALS, and HD. We validated this observation in an AD model, 4-month-old APP-transgenic (APP-tg) mouse brains and age-matched non-transgenic littermates were processed and assessed for expression levels of total CREB using immunofluorescence (IF). There appears to be a dramatic decrease in CREB expression in the APP-tg mice compared to the control littermates (Figure 7A).

We also examined CREB (total and phosphorylated) expression levels in gp120-tg mice generated by inserting the portion of the HIV-*env* gene that encodes gp120 into the mouse genome under the control of glial fibrillary acidic protein (GFAP) promoter, resulting in gp120 protein expression specifically in astrocytes (Toggas et al., 1994; Maung et al., 2014). We found that the expression level of CREB is altered in the hippocampus of these 9-month-old mice (Figure 7B). CREB decreases in the hippocampus of these mice compared to the mock suggesting that gp120 alone can result in similar effects to what is seen in AD mice.

Since CREB function depends on its phosphorylation on serine residue 133 (pCREB^S133^), we examined its expression levels. Using immunohistochemistry assay, we demonstrated that the expression level of pCREB^S133^ also decreases in the frontal cortex in gp120-tg mice compared to the Mock (Figure 7C). Quantification of pCREB^S133^ expressions in gp120-tg mice is shown in Figure 7D. Like human data, we concluded that loss of pCREB^S133^ expression triggers neurodegenerative diseases and memory impairments (Giralt et al., 2013; Middeldorp et al., 2016).

### Rolipram Restores Memory in Mice

To validate our hypothesis regarding gp120 and memory and our observations regarding gp120 and CREB, we sought to determine the effect of gp120 on CREB and spatial memory using an animal model (Thaney et al., 2018). C57Bl/6J mice received stereotaxic injections of gp120 (125 ng/μl) or saline bilaterally into the hippocampi, followed by IP injection of rolipram (1 mg/kg) or saline immediately after surgery and 36 h after surgery. Seventy-two hours post stereotaxic injection, mice underwent object location memory training. After a 24-h delay, spatial memory was tested.

**FIGURE 8:**
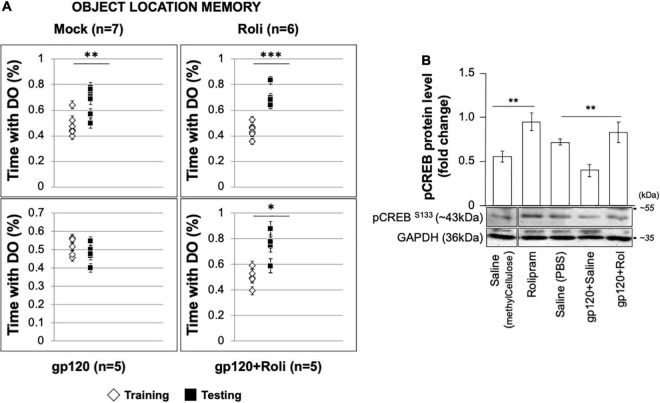
Restoring learning and memory with rolipram. **(A)** Mice were injected with either Saline [*saline (intrahippocampal) and saline (intraperitoneal); n* = *7 (5 male and 2 female)*], Rolipram [*saline (intrahippocampal) and rolipram (intraperitoneal); n* = *6 (1 male and 5 female)*], gp120 protein [*gp120 (intrahippocampal) and saline (intraperitoneal); n* = *5 (3 males and 2 females)*], or gp120 followed by rolipram [*gp120 (intrahippocampal) and rolipram (intraperitoneal); n* = *5 (3 males and 2 females)*]. Rolipram was used at 1 mg/kg concentration. 72 h later, mice were trained and tested in the object location spatial memory task. The amount of time spent exploring the object to be displaced was recorded during the training phase, and the amount of time spent exploring the displaced object during the test phase was recorded. Time spent with the displaced objects during the test phase compared to the training phase was assessed using a two-tail *t*-test as indicated. Results were judged statistically significant. **(B)** Proteins were extracted from the brain tissues (hippocampal area) of saline, gp120, and/or gp120/rolipram-injected mice and subjected to Western blot as indicated. Data represent the mean ± S.D. Results were judged statistically significant (*p < 0.05; **p < 0.01; ***p < 0.001).

Mice that received stereotaxic injections of saline control exhibited normal long-term spatial memory (measured as time spent exploring), whereas mice that received HIV_IIIB_ gp120 injections had impaired memory (Figure 8A). We found that the injection of rolipram in the presence of HIV-1 gp120 restored memory. Brains were collected from the mice and protein extracts were isolated from the hippocampal area and then subjected to western blot. As shown, the expression of phosphorylated CREB decreased in HIV-1 gp120-injected mice but maintained following rolipram injection compared to saline-injected mice (Figure 8B). These data suggest gp120-induced CREB deregulation and subsequent mitochondrial dysfunction play a role in memory impairment *in vivo.*

### Potential Mechanisms Used by gp120 Leading to Cyclic AMP Response Element-Binding Loss of Function

**FIGURE 9:**
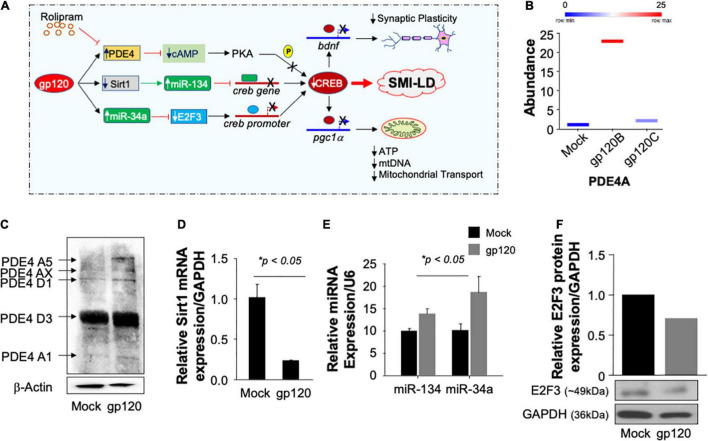
Pathways used by gp120 lead to CREB loss of function. **(A)** Schematic representation of potential pathways used by gp120 leading to CREB loss of function and development of SMI-LD. **(B)** Box plot representing the abundance of PDE4A (in duplicate) as obtained by proteomics analysis using mock and gp120-treated SH-SY5Y cells. **(C)** The protein expression of different PDE4 in SH-SY5Y cells subjected to gp120 treatment as obtained by Western blot analysis. β-actin is represented as the loading control. **(D,E)** Relative expression (done in triplicate) of Sirt1 mRNA or miR-34a and miR-134 relative to the internal controls GAPDH or U6, respectively. **(F)** The protein expression of E2F3 in SH-SY5Y cells subjected to gp120 treatment as obtained by Western blot analysis. GAPDH was used as the protein loading control.

Now that we showed that Rolipram restores CREB function and alleviates the gp120 effect, one may ask, how does gp120 protein silence CREB? According to the literature, CREB can lose its phosphorylation on serine residue 133 through several pathways (Figure 9A; Gao et al., 2010; Santerre et al., 2019; Wimmer et al., 2020). We tentatively explored these pathways, starting by examining the expression levels of PDE4 using proteomics and Western blot approaches. As shown in Figure 9B, treatment of cells with HIV_IIIB_ gp120 protein from clade B causes increased expression of PDE4A in SH-SY5Y cells compared to mock untreated or cells treated with gp120 isolated from clade C.

Similarly, treatment of the cells with gp120B protein led to increased expression of several forms of PDE4 family members as obtained by Western blot analysis (Figure 9C). We then concluded that gp120 is using PDE4 to inhibit CREB since the addition of rolipram (PDE4 inhibitor) alleviates the gp120 effect. These results also corroborate the data where the addition of rolipram restores memory impairment observed in rodents (Bourtchouladze et al., 2003; Guan et al., 2011; Zhong et al., 2016; Wimmer et al., 2020).

We also examined the ability of gp120 to activate other pathways, starting with the Sirt1 pathway. Loss of Sirt1 protein expression and function activates its target, miR-134, which in turn binds to the *creb* gene, silencing it, and causing CREB and BDNF proteins to lose functions (Shen et al., 2018). Differentiated SH-SY5Y cells were treated with 100 ng/ml of HIV_IIIB_ gp120 for 24 h. The cells were then divided into two groups where the mRNA was isolated from one group and subjected to qPCR while protein extracts were isolated from the second group and processed for Western blot. The addition of gp120 protein led to decreased mRNA expression of Sirt1 (Figure 9D) while it did increase the differential expression of miR-134 and miR34a (Figure 9E). In general, activation of miR-34a causes the suppression of its target gene *e2f3*. To validate this hypothesis, we performed a Western blot using where we observed a decreased expression of E2F3 protein (Figure 9F) leading to the conclusion that gp120 protein mainly uses the PDE4 pathway to silence CREB and also maybe the other two pathways, however, this observation needs to be determined mechanistically.

## Discussion

HIV-1 enters the brain early during the infection and evolves separately from the blood-based viral population (Lutgen et al., 2020). Available evidence suggests that HIV-associated neurocognitive disorder (HAND) is an indirect consequence of HIV-1 where viral proteins (e.g., gp120) and inflammatory mediators released by infected cells damage the neurons (Fields et al., 2016b; Canonico et al., 2022). Typical features of HAND include spatial memory impairment, inability to manipulate acquired knowledge, and generalized slowing of thought processes like symptoms observed in the elderly (Irollo et al., 2021). To examine these features, we showed the impact of HIV-1 gp120 protein on spatial memory impairment *in vitro* and *in vivo*. Looking for the mechanisms involved, we demonstrated the ability of gp120 to decrease CREB protein expression (total and phosphorylated) and function.

Our data regarding CREB’s role in spatial memory is not without a precedent. This CREB function is well studied; however, never been explored in the context of HAND. CREB binds and regulates the HIV-1 promoter activity in HeLa and Jurkat cells (Schwartz et al., 1997). It functionally interacts with HIV-Tat protein. HIV-1 Tat utilizes CREB to promote IL-10 production, though the significance of this functional interaction regarding HIV pathogenesis remains unclear (Gee et al., 2006). Several studies showed a decreased expression of CREB in neurons treated with HIV-Tat or gp120 proteins; however, CREB has never been the subject of these studies, and the mechanisms involved remain unexamined (Chun et al., 2009; Liu et al., 2018). This study presents a novel hypothesis that clarifies the relationship between gp120, CREB, and the potential role of CREB in the development of HAND.

Our data (Figures 4, 5) corroborate published studies where a decreased expression of pCREB^S133^ in the hippocampus and amygdala of aged rats contributed to the rapid loss of spatial memory (Mizuno et al., 2002; Morris and Gold, 2012; Shen et al., 2018). Also, a decrease in pCREB^S133^ expression was observed in the cerebral cortex and hippocampus of aged rats suffering from memory loss (Chung et al., 2002; Kudo et al., 2005). Similarly, aged mice subjected to contextual fear assay expressed less pCREB^S133^ and increased spatial memory impairment (Kudo et al., 2005). Another study showed somatic gene transfer of CREB protein attenuates spatial memory impairment in 15-month-old rats (Mouravlev et al., 2006). Finally, using aged mice that received the blood of young mice, an increase in pCREBS133 expression was shown, and significant progress in their spatial memory was also observed (Villeda et al., 2014). Likewise, in neurodegenerative diseases like Alzheimer’s Disease, the expression of pCREBS133 is altered (Amidfar et al., 2020).

We also observed that the addition of gp120 protein decreases the expression of PGC1α. While the relationship between CREB and PGC1α is demonstrated, the functional interaction between gp120 and PGC1α has never been examined. CREB regulates the function of PGC1α directly by binding to its promoter or indirectly by alleviating its methylation using miR-132, a CREB downstream target (Barrès et al., 2009). The absence of miR-132 activates DNMT3b that methylates the PGC1α promoter and inactivates the protein (Rahimian and He, 2016). Inactive PGC1α causes loss of mitochondrial biogenesis and energy, which explains the reduction of ATP (Figure 1) and the disappearance of mitochondrial cristae (Figure 2). In HIV-Tat-transgenic mice, activation of Tat was shown to induce the miR-132 expression and restore neurite length (Rahimian and He, 2016).

In addition to PGC1α, CREB protein regulates the brain-derived neurotrophic factor (BDNF) gene. The BDNF protein is involved in neuronal growth and neuroprotection with numerous functions in the brain. CREB and BDNF levels are low in the brains of patients affected by Alzheimer’s disease or other neurodegenerative diseases. A dearth of CREB or BDNF is associated with cognitive decline (Amidfar et al., 2020). In support of this observation, we demonstrated that gp120 decreases BDNF protein expression and shortens the neurites (Figure 3), both involved in synaptic plasticity and memory function. Several groups showed the negative impact of gp120 on BDNF protein expression and function (Bachis et al., 2012). Most of these studies focused on the role of the p75 neurotrophin receptor and linked the effect of gp120 on BDNF to this receptor (Speidell et al., 2020). However, these studies lack the interaction of CREB to CRE within the BDNF exon IV promoter in the presence of gp120. Additionally, to support previous observations regarding the loss of BDNF and its effect on synaptic plasticity, we performed experiments using gp120 protein prepared from subtypes B and C. We observed alteration of the neurites in the presence of gp120 subtype B but not C (Figure 3), which corroborate the literature that HIV-1 gp120 subtype C has less virulent effect than subtype B (Rao et al., 2014).

Additionally, the use of rolipram shed light on the understudied phosphodiesterase protein (PDE4) pathway in an HIV-1 setting. The activation of PDE4 inhibits cAMP that prevents CREB phosphorylation and binding to its cognate site (Figure 9). The addition of rolipram, a PDE4 antagonist, permits the phosphorylation of CREB, neutralizes the gp120 effect, and restores spatial memory (Figures 5, 6, 8). These results raise the possibility that gp120 could cause CREB loss of function and impaired spatial memory through activation of PDE4; however, this remains to be determined to explore which PDE4 isoform(s) is involved and the mechanisms used by gp120 leading to PDE4 activation. As described, using a selective inhibitor of PDE4 activity inhibits IL-2R expression and abolishes HIV-1 DNA nuclear import in memory T cells (Sun et al., 2000).

In addition to the PDE4 pathway, we recently demonstrated that gp120 protein activates the glycolysis pathway that affects CREB function negatively (Allen et al., personal observation). Many viruses activate this pathway and induce the mini tricarboxylic acid cycle (TCA), like KHSV, Dengue, EBV, Zika, and others (Thaker et al., 2019). Even HIV proteins, Tat and gp120, have been shown to induce the TCA pathway in neurons (Vignoli et al., 2000; Villeneuve et al., 2016; Natarajaseenivasan et al., 2018). These results led us to conclude that gp120 causes SMI-LD through two pathways where both require an active and phosphorylated CREB to prevent memory impairments.

Further, a decrease in ATP and NADH levels could also mean that gp120 reprograms the metabolism through the activation of the glycolysis pathway. To remain functional, neurons use a mini-TCA cycle that starts with the α-Ketoglutarate dehydrogenase enzyme and ends with the malate dehydrogenase enzyme. This mini-TCA cycle also utilizes tryptophan, which is a part of the kynurenine (KYN) pathway (Srivastava, 2016). KYN pathway is linked to depression and cognitive impairments in HIV-1 patients (Keegan et al., 2016). Interestingly, the conversion of KYN to kynurenine acid (KYNA) is somehow under the control of PGC1α, and loss of PGC1α promotes KYN toxicity (Wyckelsma et al., 2020). This provides additional evidence that gp120 is causing memory impairment by promoting the loss of CREB function that could affect its downstream targets like PGC1α, which could increase inflammation and depression. The downregulation of PGC1α reflects compromised mitochondrial integrity and reduced respiration. It also indicates a decrease in the density of dendritic spines in the hippocampus *in vivo* (Daum et al., 2013). Further, the glycolysis pathway promotes the accumulation of advanced glycation end products (AGEs) that prevent the cleavage of proBDNF into mature BDNF. AGEs accumulation leads to the activation of the inducible cAMP early repressor (ICER) that prevents CREB protein from interacting with its specific DNA binding site (Newbound et al., 2000) hence leading to spatial memory impairment.

Finally, one should not disregard the role of thioredoxin (Trx), a class of small proteins that acts as antioxidants and protects the mitochondria (Collet and Messens, 2010). Trx1 cleaves the disulfide bond in the V3 domain of gp120 in CD4 cells (Azimi et al., 2010) and protects mitochondria biogenesis by activating the AKT-CREB-PGC1a (Lv et al., 2013; Subramani et al., 2020). A hyperactive glycolysis pathway causes Trx1 loss of function, leads to ROS accumulation and mitochondrial respiration (Hu et al., 2018; López-Grueso et al., 2019). This pathway remains to be examined to determine whether the addition of gp120 inhibits Trx1 function.

In summary, we demonstrated that gp120 protein alters the CREB pathway and CREB downstream targets such as PGC1a and BDNF. All of which can lead to the loss of spatial memory. These results may serve to develop better therapy to prevent spatial memory loss observed in a significant number of HIV-infected patients.

## Data Availability Statement

The datasets presented in this study can be found in online repositories. The names of the repository/repositories and accession number(s) can be found below: ProteomeXchange, PXD031891.

## Ethics Statement

The animal study was reviewed and approved by the Baylor College of Medicine Institutional Animal Care and Use Committee. All experiments involving gp120-tg mice were performed per NIH guidelines and approved by the IACUC of Sanford Burnham Presbys Medical Discovery Institute. Dr. Sawaya animal protocol was approved by the Temple University IACUC.

## Author Contributions

JS, MS, CA, SA, RM, AB, and VB performed all the molecular and cellular studies. JP and JC did the stereotactic studies. JC contributed to the writing and editing of the manuscript. MK provided and performed the immunostaining of gp120-tg mice. CM and SM performed the proteomics studies. KC analyzed the proteomics data (bioinformatics). EE provided primary cultures of neurons. BS designed and directed the work and wrote the manuscript. All authors contributed to the article and approved the submitted version.

## Conflict of Interest

The authors declare that the research was conducted in the absence of any commercial or financial relationships that could be construed as a potential conflict of interest.

## Publisher’s Note

All claims expressed in this article are solely those of the authors and do not necessarily represent those of their affiliated organizations, or those of the publisher, the editors and the reviewers. Any product that may be evaluated in this article, or claim that may be made by its manufacturer, is not guaranteed or endorsed by the publisher.

## Abbreviations

ALS: Amyotrophic lateral sclerosis
BDNF: brain-derived neurotrophic factor
CRE: cAMP response elements
cART: combinatory antiretroviral therapy
CREB: cyclic AMP response element-binding protein
GFAP: glial fibrillary acidic protein
HAND: HIV-associated neurocognitive disorders
HD: Huntington’s disease
IF: immunofluorescence
IL-10: interleukin 10
mtDNA: mitochondrial DNA
OS: oxidative stress
AD: Alzheimer’s disease
PD: Parkinson’s disease
PGC1α: peroxisome proliferator-activated receptor gamma coactivator
PSD-95: post synaptic density 95
PKA: protein kinase A
PDE4: phosphodiesterase
tg: transgenic
TCA: Tricarboxylic acid cycle.
